# Human Leptospirosis on Reunion Island: Past and Current Burden

**DOI:** 10.3390/ijerph110100968

**Published:** 2014-01-10

**Authors:** Frédéric Pagès, Dominique Polycarpe, Jean-Sébastien Dehecq, Mathieu Picardeau, Nadège Caillère, Marie-Christine Jaffar-Bandjee, Alain Michault, Laurent Filleul

**Affiliations:** 1 Regional Office (Cire) of the French Institute for Public Health Surveillance (Institut de veille sanitaire), Réunion 97400, France; E-Mails: nadege.caillere@ars.sante.fr (N.C.); laurent.filleul@ars.sante.fr (L.F.); 2 Public Health Authority (Agence de santé Océan Indien ARS OI), Réunion 97400, France; E-Mails: dominique.polycarpe@ars.sante.fr (D.P.); jean-sebastien.dehecq@ars.sante.fr (J.-S.D.); 3 National Reference Centre for Leptospirosis (NRC), Institut Pasteur, Paris 75015, France; E-Mail: mpicard@pasteur.fr; 4 Biology/Microbiology/Virology/Biochemistry Units, Centre Hospitalier Universitaire (CHU North Felix-Guyon), Saint-Denis 97405, France; E-Mail: marie-christine.jaffarbandjee@chr-reunion.fr; 5 Laboratoire de Bactériologie-Parasitologie-Virologie-Hygiène, Centre Hospitalier Universitaire (CHU Sud), St. Pierre Reunion Island 97448, France; E-Mail: alain.michault@chr-reunion.fr

**Keywords:** leptospirosis, epidemiology, surveillance, human, Reunion Island, Indian Ocean

## Abstract

Since 1953, leptospirosis has been recognized as a public health problem on Reunion Island. In 2004, was implemented a specific surveillance system that included systematic reporting and the realization of environmental investigations around hospitalized cases. Here, we present the synthesis of historical data and the assessment of 9 years of leptospirosis surveillance. From 2004 to 2012, 414 hospitalized cases were reported. Cases of leptospirosis occurred mostly during the rainy season from December to May. Approximately 41% of infections occurred at home, 12% of infections occurred during aquatic leisure and 5% of cases were linked to professional activities. Furthermore, for 41% of cases, the place of infection could not be determined due to the accumulation of residential and non-residential exposure. Most of the cases of leptospirosis were linked to rural areas or traditional, rural occupations. We did not observe a shift to recreational leptospirosis as described in some developed countries. According to the new surveillance system, the number of reported cases has regularly increased since 2004. This situation is in part due to the improvement of the system in the first years but also to a real increase in the number of detected cases due to the introduction of molecular methods and to increased biological investigation into the Dengue-like syndrome by medical practitioners on the island since the Chikungunya crisis in 2006. This increase is probably due to surveillance and diagnosis biases but need to be carefully monitored. Nevertheless, the possibility of an outbreak is always present due to climatic events, such as after the “hyacinth” hurricane in 1980.

## 1. Introduction

Concerns surrounding leptospirosis infections are growing in many parts of the world, and leptospirosis is now considered an emerging neglected disease [1,2]. The burden of leptospirosis remains high in part because of the occurrence of severe forms, such as pulmonary hemorrhage syndromes, the occurrence of more frequent natural disasters (floods, hurricanes, *etc.*), and the increase in the proportion of the population living in slums [3,4,5,6,7,8,9]. 

Reunion Island is a French overseas department situated in the South West Indian Ocean near Madagascar. The temperate tropical climate has two main seasons, a rainy season during the southern summer from December to May and a dry season during the southern winter. Reunion Island has important agricultural activities dominated by sugarcane cultivation, while income from tourism is increasing as a part of the economic activities. Administratively, Reunion Island is divided into 24 municipal districts. As a French department, Reunion Island benefits from a high-level sanitary system and extensive medical coverage. 

Nevertheless, Reunion Island is not spared from leptospirosis infections [10,11]. Human infections primarily result from the *Leptospira interrogans* serogroup Icterohaemorrhagiae, which is responsible for many severe forms [12]. From 1966 to 2003, the *L. interrogans* serogroup Icterohaemorrhagiae accounted for 70% to 83% of all hospitalized cases [13]. All past studies showed a significant predominance of males with severe infections and a high prevalence of pulmonary manifestations. Additionally, cases occur throughout the year but primarily during the rainy season and in the rainiest areas of the island [12,13,14,15,16,17]. Therefore, the majority of the identified leptospirosis cases were severe and required hospital care. From the 1970s to the 2000s, 13% to 17% of severe cases resulted in lethality [11,12]. Nevertheless, mild forms also exist, as highlighted by a study conducted in 1987, which showed the same seroprevalence in males and females, suggesting that females were also exposed to *Leptospira spp* but did not develop severe forms of the disease [18]. The animal reservoir is diverse, including endemic or non-endemic and wild or domestic mammals [19,20]. The black rat, *Rattus rattus*, abundant in most regions and biotopes of the island, is considered as the primary reservoir and transmitter of *Leptospira spp* in the environment [21]. The role of stray dogs, also abundant in the island, remains unclear. 

Historically, the first outbreak of leptospirosis on Reunion Island most likely occurred in 1868, but the first report of confirmed cases was not reported until 1953 (11 cases: *i.e.*, four cases per 100,000 inhabitants) [22]. Because leptospirosis has been recognized as a public health problem on the island, many studies have been conducted to assess the burden of leptospirosis over the years [23,24,25,26,27]. Because the reporting system has changed many times and according to the source of data (systematic reporting of the French national health authorities from 1965 to 1985, punctual reports of the regional health authorities in the early 2000s, reports of the French National Reference Center (NRC) for leptospirosis from 1975 to 2004 and punctual surveys using hospital data), the real burden of leptospirosis is very difficult to assess retrospectively [12,13,14,15,16,17]. From 1966 to 1974, the mandatory notification of cases resulted in an average reported incidence of 4.3 cases per 100,000 inhabitants per year. From 1975 to 1985, when two sources of data were available, the average incidence varied from 7.5 to 13 cases per 100,000 inhabitants. For 1982 only, the incidence varied from 7.4 to 21.3 cases per 100,000 inhabitants. From 1987 to 1997, no data were available from the literature, the regional health agency reports or the NRC reports. From 1998 to the early 2000s, as the number of strains sent to the NRC was increasing, two studies were conducted to assess the burden of leptospirosis: a descriptive study in 2002 and a case control study in 2003 to precisely identify the determinants of leptospirosis on the island [28,29]. In the case control study, two analyses were conducted. The first analysis considered the whole survey population, and the factors associated with leptospirosis in multivariate analysis were as follows: belonging to a high-risk profession (OR = 14.1 [3.6–54.9]), fishing or hunting (OR = 6.6 [1.7–25.6]), contact with wild fauna (mainly rats) (OR = 5.3 [1.4–20.4]), and participating in rural leisure (essentially hiking) (OR = 3.0 [1.1–7.9]). The second analysis considered workers and non-workers separately in two multivariate analyses. For workers, the factors associated with leptospirosis were as follows: belonging to a high-risk profession (OR = 22.4 [3.4–145.5]) and having contact with poultry (OR = 11.2 [1.5–83.9]). For non-workers (retirees, students, and the unemployed), the factors associated with leptospirosis were as follows: Fishing or hunting (OR = 9.6 [2.2–43.2]), participating in rural leisure (essentially hiking) OR = 3.7 [1.2–11.4]) and gardening (OR = 3.4 [1.1–10.8]). The average incidence for 2002-2003 was 6.8 cases per 100,000 inhabitants. Then, in 2004, the decision to implement a regional surveillance system (including systematic reporting and environmental investigations around hospitalized cases) for leptospirosis was made on the island. We present here the results from nine years of leptospirosis surveillance from 2004 to 2012. 

## 2. Materials and Methods

### 2.1. Leptospirosis Surveillance

The regional office of the French Institute for Public Health Surveillance (Cire OI) performs surveillance of leptospirosis on Reunion Island. The study population included all people living on Reunion Island (837,868 inhabitants in 2012). Only leptospirosis infections acquired on Reunion Island by residents were considered, and thus, infections contracted overseas by residents or tourists were excluded. For each suspected leptospirosis case, the practitioner who saw the patient or the biologist who conducted the biological analysis (DNA amplification by Polymerase Chain Reaction (PCR) in blood or urine, ELISA IgM, and/or Microscopic Agglutination Test (MAT)) filled out a form that was sent to the health watch platform (CVAGS) of the French Regional Health Agency for the Indian Ocean (ARS OI). The forms were then transmitted to the Cire OI for the data to be analyzed. A medical form providing information concerning the patient, clinical symptoms, outcome, and biological diagnosis was completed by the practitioner following admittance of the patient. Practitioners who sent an incomplete form were contacted by the CVAGS for data completion. Cases were classified on the basis of case definitions that included clinical and/or biological data. Two types of cases were considered: confirmed cases (positive cultures, positive PCR from blood or urine, or positive MAT on a single serum with titer >1/400) and possible cases (clinical manifestations compatible with leptospirosis (fever >38.5 °C with arthralgia and myalgia) and positive ELISA IgM). Cases that did not fit the definition (lack of biological proof, isolated positive ELISA IgM) and non-hospitalized cases were excluded. After the case was validated, an environmental study was conducted for each case. The ARS agents questioned the patients at home about their habits, the use of protective measures, and their possible risk factors (residential exposure, professional exposure or leisure exposure in the 30 days before the first signs) to identify the place of contamination. The agents provided health education and information about leptospirosis transmission during their interview. In addition, environmental investigations were also conducted for non-hospitalized patients, and environmental forms were completed.

### 2.2. Analysis

The study analyzed data from the 2004–2012 period. Data from the medical and environmental forms were recorded using EPIData 3.1 ^®^ and analyzed with Stata 11 ^®^ statistical software using the chi-squared test for observed frequencies and the t-test for quantitative data. A multiple correspondence analysis was conducted to describe the population of the hospitalized cases according to their identified exposures to leptospirosis. Then, an agglomerative clustering made on variable modalities was performed to describe specific high-risk groups of people. Demographic data from the French Institute of Statistic and Economic Studies (INSEE) were used to calculate the incidence rates by year, as well as within different periods of study on the island and in the 24 communal districts. Using the regional database PMSI (Programme Medicalise des Systemes d’Informations), an estimation of the annual hospital cost of leptospirosis was made using the related cost of the GHS (homogeneous group of stays) including leptospirosis and the mean per day cost of an intensive care unit (ICU) stay.

Data from the French NRC for leptospirosis were used to estimate the infecting serogroup on Reunion Island. The NRC receives serum samples from mainland France and French overseas territories (approximately 4,500 samples each year) to perform the serological diagnosis of leptospirosis by MAT and ELISA IgM. The NRC also collects the diagnosis data from other laboratories. For Reunion Island, the diagnostic data include the PCR data from the university hospital Félix Guyon (Saint-Denis Réunion) and the PCR and MAT data from the university hospitals of Groupe Hospitalier Sud Réunion (Saint Pierre), as well as, to a lesser extent, the private French laboratories. Annual reports from the NRC can be found in the NRC and Pasteur Institute websites [14,30].

## 3. Results

### 3.1. Leptospirosis Incidence and Trends according to the Surveillance System

**Figure 1:**
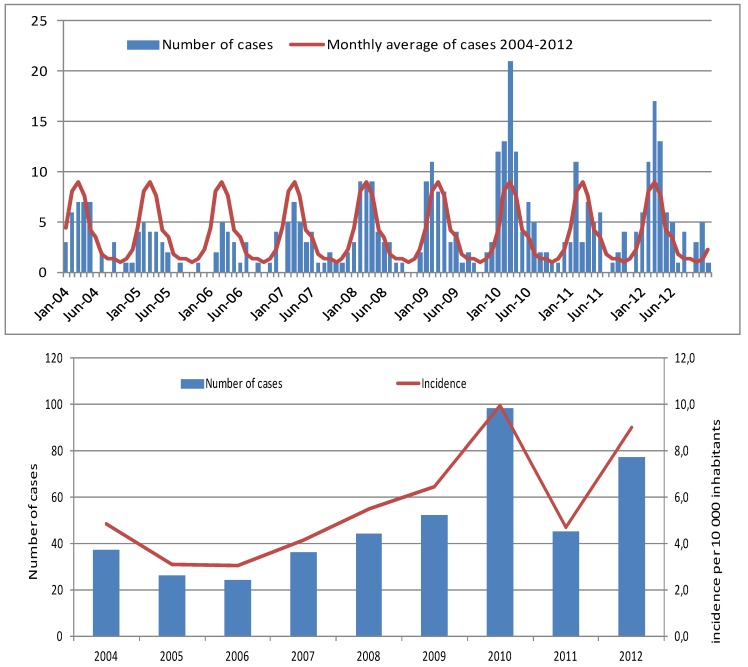
(**a**) Monthly repartition of the 414 cases of leptospirosis reported on Reunion Island from 2004 to 2012. (**b**) Evolution of the incidence and the incidence rate of Leptospirosis on Reunion Island from 2004 to 2012.

From 2004 to 2012, 414 hospitalized cases of leptospirosis were reported by the regional surveillance system, but data were available for only 405 cases (nine files were missing from the 2004 to 2005 surveillance). Figure 1a presents the monthly repartition of these cases. The number of cases by month is clearly directly linked to the season. Cases of leptospirosis occurred throughout the year, but most of the cases were reported during the rainy season from December to May. 

**Figure 2:**
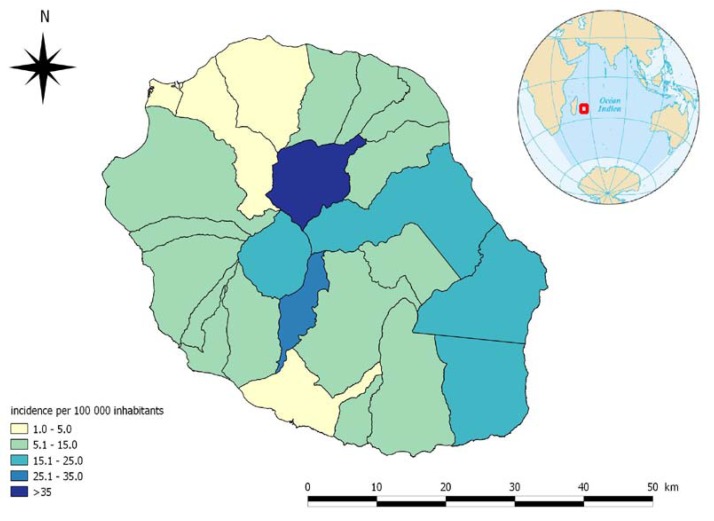
The change in the average incidence of leptospirosis on Reunion Island by commune between 2008 and 2012 is shown.

In Figure 1b, the evolution of the incidence and the incidence rate from 2004 to 2012 is shown. The incidence rate varies from 3.1 cases per 100,000 inhabitants in 2005 to 10 cases per 100,000 inhabitants in 2010. The mean annual incidence, calculated from 2004 to 2012, was 5.6 cases per 100,000 inhabitants. During this period, this rate varied by communal district from 2.3 cases per 100,000 inhabitants in Le Port (an industrial district on the border of the seas situated in the driest area of the island) to 54.7 cases per 100,000 inhabitants in Salazie (a rural district in the highlands of the island situated in the rainiest part of the island). The average incidence rates in the communes from 2008 to 2012 are represented in Figure 2. According to the surveillance, after a decrease in leptospirosis incidence from 2004 to 2006, the number of cases has steadily increased since 2007. The average lethality rate for 2004 to 2012 was 8% (20 deaths) but fell to 4% when data from 2006 (lethality rate of 38%, year of the massive Chikungunya outbreak on Reunion Island) were excluded. In 2006, many leptospirosis cases were initially clinically considered to be Chikungunya cases. Delays in consultation, diagnosis and care were most likely the reason for the high lethality [31]. 

### 3.2. Utilization of the Data Obtained from the Medical Forms

**Figure 3:**
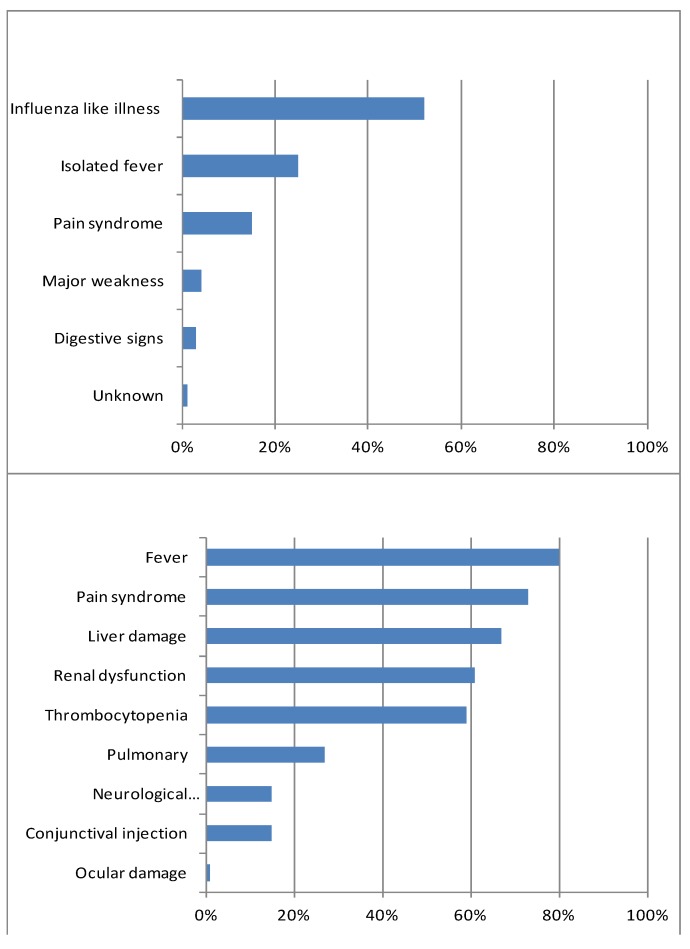
The distribution of the first symptoms described by leptospirosis cases, and the distribution of clinical manifestations of leptospirosis reported by medical practitioners from 2004 to 2012 on Reunion Island.

The distribution of the first symptoms described by inpatients at the beginning of their disease and the clinical manifestations reported by medical practitioners are shown in Figure 3. Hospitalization and ICU data were available for 405 inpatients from 2004 to 2012. Of these 405 inpatients, 142 (35%) required ICU treatment. 

### 3.3. Hospitalization Costs

From 2004 to 2012, the mean cost of hospitalization for leptospirosis was 6,344 Euros, which amounted to a total cost of approximately 4.6 million Euros for all hospitalized cases of leptospirosis during this period.

### 3.4. Identification of Leptospira Serogroups Involved in Human Leptospirosis Infections on Reunion Island

From 2004 to 2012, the NRC received serum samples or collected diagnosis data for 629 leptospirosis cases on the island, including 267 cases that were diagnosed by PCR. MAT analyses were available for 338 cases, and the serogroup Icterohaemorrhagiae was found in 194 (57.4%) cases. Other samples included primarily serum taken very early in the disease, which showed a high degree of cross-reactivity between different serogroups as previously described in acute-phase samples [32]. 

### 3.5. Utilization of the Data Obtained from the Environmental Forms

**Figure 4:**
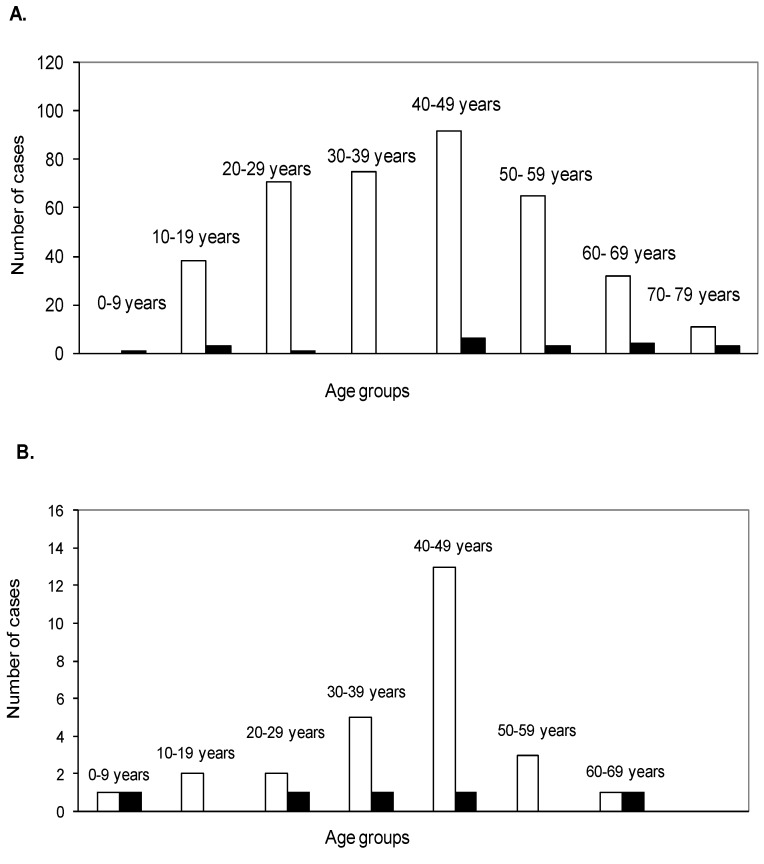
The distribution of cases by age and sex (women are shown in black columns and the men are shown in white columns) for the 405 hospitalized (**a**) and 32 non-hospitalized (**b**) cases reported from 2004 to 2012 on Reunion Island.

Environmental data were available for 405 hospitalized cases (385 confirmed cases and 20 possible cases) and 32 non-hospitalized cases (26 confirmed cases and six possible cases). 437 infections occurred in men (384 hospitalized and 27 non-hospitalized) and 26 in women (21 hospitalized and 5 non-hospitalized). The environmental investigations were conducted for 350 cases: 319 hospitalized cases (309 confirmed cases and 10 possible cases) and 31 non-hospitalized cases (30 confirmed cases and one possible case). The total number of investigations that were not conducted was 88 (77 confirmed cases and 11 possible cases). The primary reason investigations were not conducted was the refusal of the patients or their families. The distribution of leptospirosis cases by age and sex is presented in Figure 4. There were no statistically significant differences in the ages between men and women as well as between hospitalized and non-hospitalized cases. In total, 6.5% of non-hospitalized cases were children (age < 15 years), while only 1.5% of hospitalized cases were children, but this difference was not statistically significant. However, the proportion of women was significantly higher in non-hospitalized cases compared to hospitalized cases (18.5% *vs.* 5%, *p* < 0.05, Fisher’s exact test). 

Using demographic data, we compared the evolution of the incidence by age group between the mid-sixties and the 2004–2012 periods. The incidences of leptospirosis varied for all age groups: From 0.75 to 1.6 cases per 100,000 inhabitants for people under 20 years old, 24.4 to 22.7 cases per 100,000 inhabitants for people 20 to 59 years old and 26 to 32 cases per 100,000 inhabitants in people over 60 years old.

There was no difference observed for the prevalence of risk factors between hospitalized and non-hospitalized cases. However, the prevalence of residential, professional and leisure risks were calculated for all 350 cases that were investigated at home. According to their declarations, 5.4% of cases were retirees, 7.1% were students, 38.4% were working and 49.1% were unemployed. Among working people, 86% belonged to a leptospirosis high-risk profession. Among unemployed people, 69% gardened regularly. Residential risks were present in 80% of cases, professional risk in 36% and leisure risks in 53%. The description of the different risks is presented in Table 1.

**Table 1:** The distribution of residential, professional and leisure risks in 350 leptospirosis cases, Reunion Island, 2004–2012.

Residential Risks		Leisure Risks		Professional Risks **	
At least one animal at home	80.2%	Gardening	66,4%	Farmers	49.6%
Living in a rural area	69.6%	Breeding at home *	45.4%	Green space workers	15.5%
Rats in the house	38.4%	Fishing	20.5%	Construction workers	9.3%
Rats in the neighborhood	31.4%	Bathing	24.3%	Other at risk professions	8.6%
Poor housing conditions	20.0%	Hunting	7.6%	Professions not at risk	17.0%
Live in a floodable area	10.6%				
Use untreated water	7.2%				

Notes: * mainly poultry. ** of the employed people (38,4% of cases).

Before clinical manifestations of the disease, wounds or skin abrasions were present in at least 28.6% of cases: 49.4% before the suspected exposure to *Leptospira spp* and 50.6% acquired during the suspected exposure. During their activities, 59.6% of cases did not use protective wear (boots, gloves, boots dressing, *etc.*), 24.8% used incomplete protection (e.g. boots without gloves), and 4.4% used complete protection at work but none at home or during leisure activities. 

Some cases reported multiple exposures, but the classification of residential, professional or leisure risks allowed for the determination of the probable place of infection for 59% of cases: 41% of infection occurred at home, 12% of infection occurred during aquatic leisure and 5% of infection was directly linked to professional activities. Nevertheless, for 41% of cases, the place of infection could not be determined due to the accumulation of residential and non-residential exposures. 

A multiple correspondence analysis was performed (data not shown). However, the two main axes of analysis could explain less than 20% of the observed variability but distinguished between people belonging to a high-risk profession and showed a possible correlation between young age and the practices of bathing, fishing and hunting. 

In addition, agglomerative clustering permitted different groups of individuals to be identified. The first group (women and people over 60 years of age) included populations that were not significantly affected by leptospirosis. Meanwhile, this clustering highlighted the following groups at higher risk: People belonging to a high-risk profession, people under 20 years of age that participate in aquatic activities, people between 20 and 30 years of age that fish and people between 50 and 60 years of age that garden at home. 

## 4. Discussion

From 2004 to 2012, there was an increase in the number of reported leptospirosis cases to the regional surveillance system. This phenomenon occurred in part due to improvements in the system compared to the reports of the French NRC for leptospirosis [14]. The discrepancy between the surveillance and the NRC data occurred mainly during the first few years following implementation of the surveillance system. Since 2008, the data from both sources have been similar. While the surveillance system may underestimate the incidence of leptospirosis resulting in hospitalization, the trends in this disease over the years are well estimated and the findings of the regional surveillance are now reliable. Nevertheless, the surveillance system is focused on hospitalized cases and is most likely underestimating the number of ambulatory cases. The number of non-hospitalized patients could be higher, such as in 2010 when the number of cases determined by the NRC was higher than the number of hospitalized cases and the number of reported cases (125 *vs.* 101 and 98, respectively). The surveillance of hospitalized cases is also most likely underestimating the weight of leptospirosis because it is focused only on severe forms caused by the serogroup Icterohaemorrhagiae. From 2004 to 2012, there was no way to distinguish between hospitalized and non-hospitalized cases of leptospirosis in the samples sent to the NRC. The apparent decrease in the serogroup Icterohaemorrhagiae on the island from 2004 to 2012 compared to previous years should be cautiously interpreted. One possible explanation is the increase in the use of PCR in hospitals, which is associated with a decrease in the use of MAT and results in difficulties obtaining a second serum sample for many hospitalized patients. Another explanation is a diagnosis bias resulting from increased biological investigation into the Dengue-like syndrome by medical practitioners on the island since the Chikungunya crisis in 2006. Mild forms of leptospirosis caused by a serogroup other than Icterohaemorrhagiae were unexplored in previous years and are potentially diagnosed more frequently now. 

From 2008 to 2012, the mean annual incidence according to the surveillance is 8.2 cases per 100,000 inhabitant, which is approximately twenty times higher than in continental France (0.43 cases per 100,000 inhabitants) [14]. When compared with the other islands in the southern West Indian Ocean for which recent data are available, the burden of leptospirosis is higher than in the “twin” island of Mauritius (2.5 cases per 100,000 inhabitants) but lower than in Mayotte (54 cases per 100,000 inhabitants) [33,34]. Nevertheless, the lethality remains high: 4% on Reunion Island in recent years despite the density and the high quality of the sanitary system *vs.* 0.7% in Mayotte [33]. This situation is most likely due to the predominance of hospitalized cases with the serogroup Icterohaemorrhagiae, which may be responsible for more severe forms of the disease on Reunion Island. The surveillance system uncovered a high level of pulmonary involvement (27%), which was already described on the island [27]. Without early detection made possible by the extended use of PCR and the vigilance of clinicians, the burden would most likely be higher as was observed during the Chikungunya outbreak of 2006 when the lethality reached 38% because of delayed diagnosis [31]. 

The situation of leptospirosis is very different depending on the year, season and district. The transmission is higher during the rainy season as already described and is more intense in years with heavy rains, such as in 2010 and in the rainiest districts of the east and the center of the island [35]. In those parts of the island, the incidence of leptospirosis is comparable to the hottest spots in the world [36].

Nevertheless, according to clinicians, the burden of leptospirosis has drastically decreased since the 1970s. This decrease in incidence is congruent with the decrease in seroprevalence from 1.1% to 0.66% in 1987 to 2006, respectively, in the general population [37]. However, this decrease in leptospirosis burden is not clearly evident when looking at surveillance data between the 1960s and today because of underreporting but also due to the progress made in biological diagnosis, including molecular biology tools that now permit the detection or confirmation of infection more easily enabling the reporting of more cases. 

On Reunion Island, leptospirosis infections are highest in adult males. Similar to what has been described since the 1960s, 95% of hospitalized cases were male in our survey. Meanwhile, women and children were more numerous among non-hospitalized cases, suggesting that women and children contract milder forms of leptospirosis. This finding is compatible with the results of the 1987 survey, which found similar seroprevalence rates in both males and females. An excess of male cases are often retrieved in leptospirosis surveillance data. Traditionally, this has been explained through different occupational/recreational exposures, but some studies found higher severity and higher leptospiremia in males than in females. However, it is possible that disease susceptibility and clinical outcomes could be different according to sex and that the surveillance system may more easily capture severe cases. Therefore, the increase in the number of male cases compared to female cases could be in part due to a surveillance bias [38]. 

Only 1.5% of hospitalized cases were under 15 years old. When describing the current repartition by age group, leptospirosis is clearly more prominent in adult people. People aged 20 to 59 years old accounted for 53% of the population but represented 77% of cases. Conversely, people less than 20 years old accounted only for 10.4% of cases but represented 34.9% of the population. Meanwhile, the proportion of people over 60 years old was similar between the number of cases and the whole population (12.3% *vs.* 11.3%). In the 1960s, people ages 20 to 59 years old accounted for 88% of all cases but they represented only 38.4% of the total population, while people under 20 years of age represented 56.3% of the population but accounted only for only 7% of cases. Additionally, people over 60 years old represented 5% of the cases and 5% of the total population. The mean age of cases in 2004–2012 was 40 years for men and 46.5 years for women, compared to 35 years for men and 38 years for women in the 1960’s. This difference is in part due to aging of the island’s population but also because of the decrease in the number of cases in the 20- to 30-year-old age group, which accounted for 44% of cases in the 1960s compared to 17% today (*p* < 10^−5^). Because this age group has always increased in the whole population since this period, the change in leptospirosis incidence among the different age groups is most likely not only due to the population aging but also results from modifications in the population’s behaviors and exposures. For example, walking barefoot was very common 50 years ago but has virtually disappeared today. Additionally, the use of rivers to bathe and wash clothes has since been forbidden. No data are available to directly assess the decrease in exposures, but the fight against the slums began in the 1970s and the progress in water sanitation and the increasing urbanization has most likely decreased exposures to a large part of the population. The incidence of leptospirosis has doubled for people less than 20 years of age. This could correspond to the second group at risk identified by the agglomerative clustering analysis: people less than 20 years old that participate in aquatic activities. The incidence decreased slightly in people 20 to 59 years old and increased in people more than 60 years old. This could be explained by the diminution in new generations of the traditional at risks practices and their persistence in the elderly. In fact, when looking at the risk factors identified around cases, some have not changed in prevalence since the 1960s (living in a rural area, the presence of rodents, farming and rearing at home, belonging to a high-risk profession) and others are always present, such as bathing or fishing in a river, which could potentially account for a higher number of cases in an area [13,15,25]. A residential risk is currently present for 80% of cases, and most of those people are not using any protective devices at home against leptospirosis. Therefore, it is very difficult in most of cases to identify the place and the mode of contamination. If most contamination occurs because of professional or recreational farming, other exposures are most likely occurring but their importance is difficult to evaluate due to the high frequency of residential or agricultural exposure. Thus, cases have occurred in local or tourist hikers without any other risk factors. 

Nevertheless, most of the cases report leisure or professional agricultural activities and live in a rural environment. Most infections occur in people that do not use any protection during their farming activities. The simple use of mechanical protections during agricultural activities or the protection and disinfection of wounds could most likely prevent most leptospirosis cases on the island. Furthermore, on Reunion Island, the role of *Rattus spp.* as a reservoir and disseminator of serogroup Icterohaemorrhagiae has been highlighted [20]. The presence of rats in the environment is described by most cases at home or during leisure or professional activities. Rodent control activities have to been strengthened and coordinated at the island level. Additionally, social mobilization activities have to be implemented to enhance the participation of the community in these rodent control activities.

## 5. Conclusions

Leptospirosis is always of concern on Reunion Island. Most cases are currently linked to rural areas or agricultural activities. Recreational exposure is most likely not as important as described in some developed countries. According to different sources of data, the leptospirosis burden has decreased since the 1970s to 2000s. According to the new surveillance system, the number of reported cases has regularly increased since 2004. This situation is in part due to the improvement of the system in the first years but also to a real increase in the number of detected cases due to the introduction of molecular methods and to increased biological investigation into the Dengue-like syndrome by medical practitioners on the island since the Chikungunya crisis in 2006. This increase is probably due to surveillance and diagnosis biases but need to be carefully monitored. Nevertheless, the possibility of an outbreak is always present due to climatic events, such as those after the “hyacinth” hurricane in 1980 or due to the increase in frequenting rivers or basins after the removal of restrictions on aquatic activities in the sea due to recent sharks’ attacks [26]. The surveillance system is reliable, but it could be easily improved if it records all cases occurring on the island regardless of hospitalization status. When available, current serogroup data confirm the predominance of Icterohaemorrhagiae in severe forms since the first study was conducted in the 1970s. However, the isolation of clinical *Leptospira* strains is rare due to the fastidious growth in culture of this species. In addition, PCR has in recent years been increasingly used for the diagnosis of leptospirosis, replacing MAT. Thus, we have little recent information on circulating strains on Reunion Island. The emergence of new serotypes has to be considered. The identification of the circulating strains on Reunion Island will help establish appropriate control and preventive measures on this island where the disease is endemic.
